# Using bacterial inclusion bodies to screen for amyloid aggregation inhibitors

**DOI:** 10.1186/1475-2859-11-55

**Published:** 2012-05-03

**Authors:** Anna Villar-Piqué, Alba Espargaró, Raimon Sabaté, Natalia S de Groot, Salvador Ventura

**Affiliations:** 1 Departament de Bioquímica i Biologia Molecular, Facultat de Biociències, Universitat Autònoma de Barcelona, E-08193, Bellaterra, Spain; 2 Institut de Biotecnologia i de Biomedicina, Universitat Autònoma de Barcelona, E-08193, Bellaterra, Spain; 3 Present address: Departament de Fisicoquímica, Facultat de Farmàcia, Universitat de Barcelona, Avda. Joan XXIII, 08028, Barcelona, Spain; 4 Present address: Medical Research Council Laboratory of Molecular Biology, Hills Road, Cambridge, CB2 0QH, United Kingdom

**Keywords:** Amyloids, Inclusion bodies, Protein folding, Protein aggregation, Metals, Alzheimer

## Abstract

**Background:**

The amyloid-β peptide (Aβ42) is the main component of the inter-neuronal amyloid plaques characteristic of Alzheimer's disease (AD). The mechanism by which Aβ42 and other amyloid peptides assemble into insoluble neurotoxic deposits is still not completely understood and multiple factors have been reported to trigger their formation. In particular, the presence of endogenous metal ions has been linked to the pathogenesis of AD and other neurodegenerative disorders.

**Results:**

Here we describe a rapid and high-throughput screening method to identify molecules able to modulate amyloid aggregation. The approach exploits the inclusion bodies (IBs) formed by Aβ42 when expressed in bacteria. We have shown previously that these aggregates retain amyloid structural and functional properties. In the present work, we demonstrate that their *in vitro* refolding is selectively sensitive to the presence of aggregation-promoting metal ions, allowing the detection of inhibitors of metal-promoted amyloid aggregation with potential therapeutic interest.

**Conclusions:**

Because IBs can be produced at high levels and easily purified, the method overcomes one of the main limitations in screens to detect amyloid modulators: the use of expensive and usually highly insoluble synthetic peptides.

## Background

In the last few years, protein aggregation has emerged from a neglected area of protein chemistry as a transcendental issue in biological and medical sciences, mainly because the deposition of proteins into insoluble amyloid fibrils is being found behind an increasing number of human diseases such as Alzheimer’s disease (AD) or type II diabetes [1-4]. Therefore, there is an increasing interest in developing methods to identify molecules that trigger the aggregation of these proteins inside the organism as well as to discover chemical compounds that can interfere with these pathways, having thus therapeutic potential [5-7].

The pathological hallmark of AD is brain deposition of amyloid plaques composed predominantly by the Aβ42 peptide isoform [8-10]. The origin of these insoluble extracellular neurotoxic deposits is still not completely clear, and multiple factors such as pH, peptide concentration, oxidative stress and metal ions have been reported to trigger their formation [11,12]. Here we present a fast, cost-effective high-throughput approach to study conditions and molecules that affect Aβ42 aggregation. The assay is based on the use of the inclusion bodies (IBs) formed by an Aβ42-GFP fusion protein in bacteria. IBs formation has long been regarded as an unspecific process relaying on the establishment of hydrophobic contacts [13,14]. However, there are now strong evidences demonstrating that bacterial IBs formation shares a number of common features with the formation of the highly ordered and pathogenic amyloid fibrils linked to human diseases [15-18]. Therefore, IBs have become an attractive model to study protein aggregation and their consequences in simple but biologically relevant environments [19-21]. IBs are formed inside the cell when the folding of proteins into native conformations is competed by a faster establishment of anomalous intermolecular interactions that leads to the formation of insoluble aggregates [22]. In the present work, we exploit this kinetic competition *in vitro* to screen for compounds that can modulate protein aggregation. As a proof of principle, we demonstrate the ability of the approach to detect the effect of metal ions on Aβ42 aggregation as well as to identify compounds that block this metal-induced reaction.

## Results and Discussion

### Refolding Aβ42-GFP IBs is sequence specific

**Figure 1:**
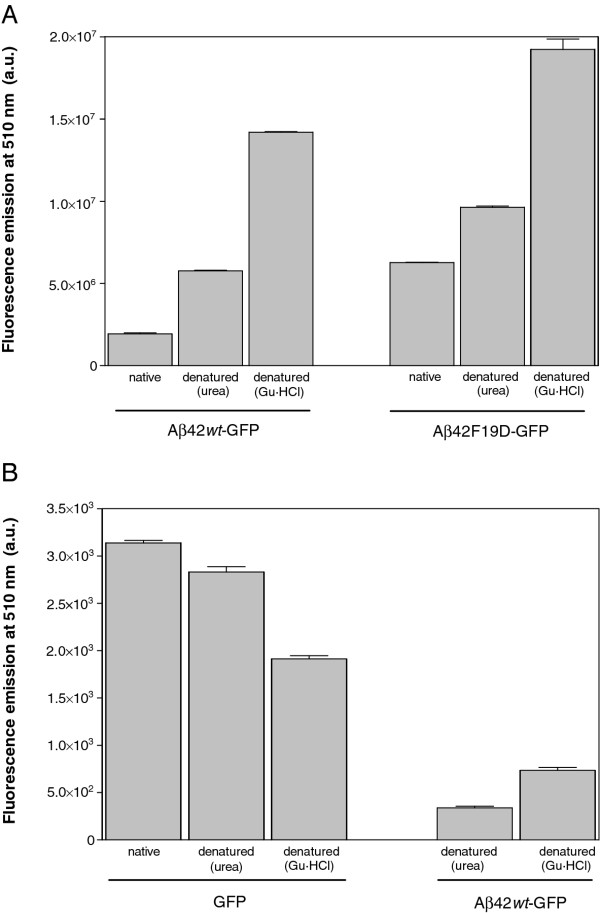
**Fluorescence recovery after denaturation. (A)** Purified IBs were incubated in PBS in the absence (native) and presence of 8 M Gu·HCl or 10 M urea for 4 h and diluted 100-fold in PBS. **(B)** Purified untagged GFP (left) and IBs (right) were incubated in PBS in the absence (native) and presence of 8 M Gu·HCl or 10 M urea for 4 h and diluted 100-fold in PBS. In all cases, after incubation for 16 h fluorescence was recorded at 510 nm.

We have previously shown that the IBs formed by Aβ42 display amyloid-like properties whether the peptide is expressed alone [23] or fused to fluorescent proteins [16,24]. We have constructed a set of 20 different Aβ42–GFP variants, which differ only in a single residue in the peptide’s central hydrophobic region [25]. All these proteins are expressed at similar levels in *E. coli* and form insoluble IBs [25]. Nevertheless, the fraction of active GFP in those aggregates is significantly different (Figure 1). The IBs fluorescence correlates with the aggregation propensity of the specific Aβ42 mutant [26]. This correlation is the result of a kinetic competition between the folding of the GFP domain and the aggregation of the fusion protein, which is driven by the Aβ42 moiety. Therefore, the slower the fusion protein aggregates, the higher the IB fluorescence emission is and *vice versa*. In this way, IBs fluorescence reports on intracellular aggregation kinetics [22,26,27].

We wondered if the kinetic competition between GFP folding and Aβ42 aggregation can be reproduced *in vitro.* To this aim we used the IBs formed by the *wild-type* peptide fusion (Aβ42*wt*-GFP) and the F19D mutant (Aβ42F19D-GFP), which display the highest and lowest aggregation propensities in our library, respectively [22]. Purified IBs were denatured to remove the polypeptide contacts supporting the aggregate structure. This provides unfolded and isolated species for the subsequent *in vitro* refolding step and guarantees that all inter- or intra-molecular contacts are established *de novo* as it happens after protein synthesis in the cell. IBs were chemically denatured using two chaotropic agents, 10 M urea and 8 M Gu·HCl. Each unfolded Aβ42-GFP fusion was diluted in refolding buffer and the amount of recovered active GFP monitored using fluorescence spectroscopy (see Methods). The same conditions were used to unfold and refold equimolar concentrations of native untagged GFP. As it can be seen in Figure 1A, independently of the IBs peptide variant, the level of recovered GFP activity was higher when Gu·HCl was used as denaturant. This is in contrast with the results obtained with untagged GFP, for which denaturation with urea resulted in higher fluorescence recovery (Figure 1B), suggesting that the used denaturant might affect the aggregation/refolding pathway. The proportion of fluorescent GFP recovered after refolding was always higher than that in the original IB (Figure 1A). Aggregation usually corresponds to a second or higher order reaction and therefore, aggregation rates are extremely dependent on protein concentrations [28]. Since the protein concentrations used during *in vitro* refolding are much lower than those existent *in vivo,* the folding of the GFP domain can compete more efficiently with the aggregation process, providing a larger dynamic response than in bacteria. However, the refolding efficiency of Aβ42-GFP IBs is about ~10-fold and ~4-fold lower than this of untagged GFP after denaturation in urea and Gu·HCl, respectively, suggesting that, as it happens *in vivo*, the aggregation of the Aβ42 moiety competes the folding of GFP. Importantly, the activity recovery from the mutant IBs is higher than that from IBs formed by the *wild-type* sequence, supporting a kinetic competition between GFP folding and Aβ42 aggregation *in vitro.* The predicted lower aggregation rate of the mutant would account for the higher fluorescence recovery. By analogy, any agent that would increase the intrinsic aggregation rate of Aβ42 will decrease the final amount of functional GFP and *vice versa,* allowing to screen for promoters or inhibitors of the protein aggregation process.

### Detection of the Aβ42 aggregation-promoting effect of ionic metals

**Figure 2:**
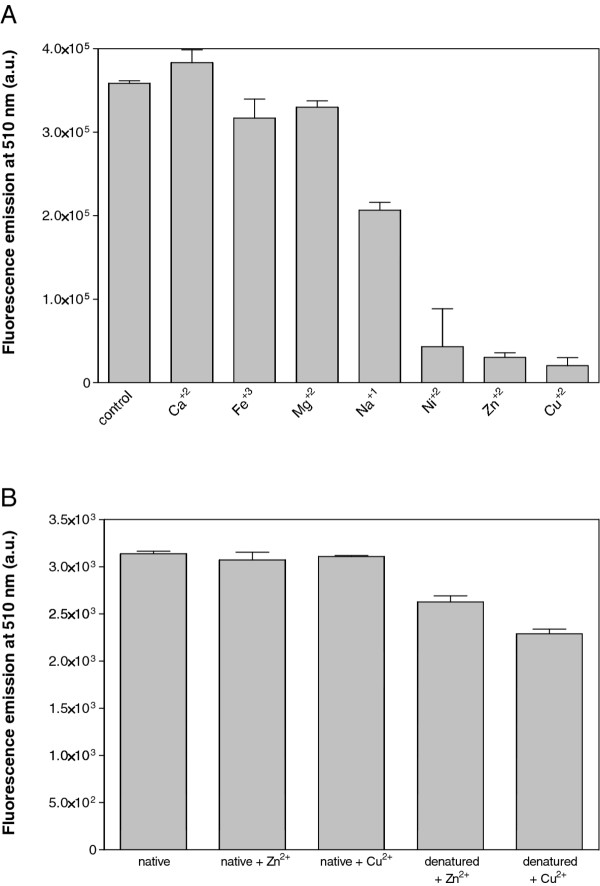
**Fluorescence recovery in the presence of metallic ions. (A)** Aβ42*wt*-GFP IBs were denatured in 8 M Gu·HCl for 4 h and diluted 100-fold in PBS (control) or in PBS containing different metallic ions at 25 μM final concentration. **(B)** Purified untagged GFP and IBs were incubated in PBS in the absence (native) and presence of 8 M Gu·HCl for 4 h and diluted 100-fold in PBS containing Cu^+2^ and Zn^+2^ at 25 μM final concentration. In all cases, after incubation for 16 h fluorescence was recorded at 510 nm.

**Figure 3:**
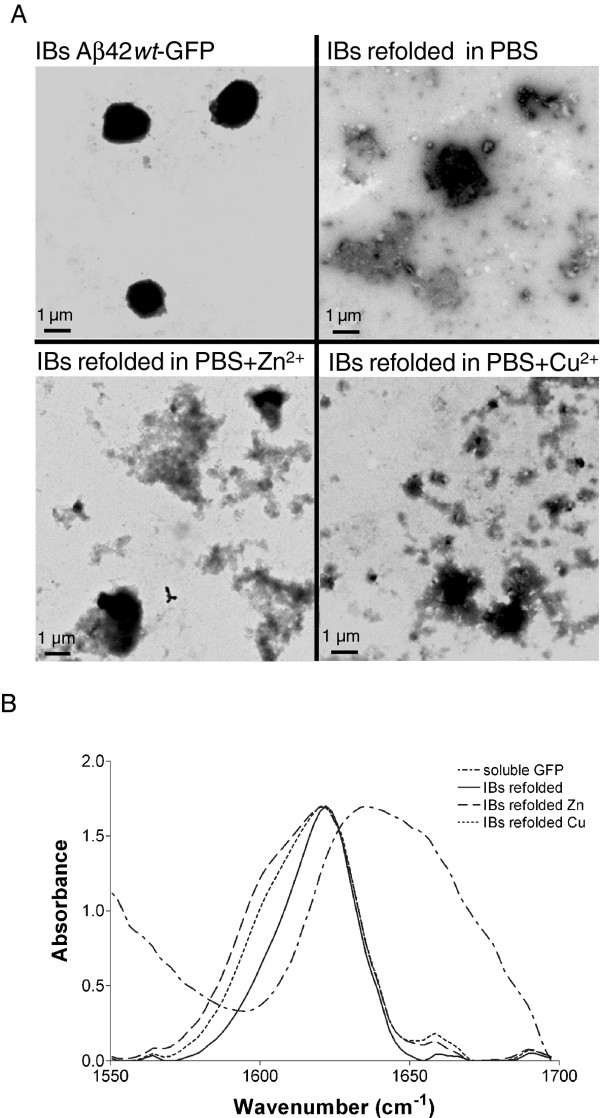
**Morphology and secondary structure of aggregates. (A)** Morphology of purified IBs and aggregates in refolding solutions in the absence and presence Cu^+2^ and Zn^+2^. **(B)** Analysis of the secondary structure of aggregates in refolding solutions in the absence and presence Cu^+2^ and Zn^+2^ by FT-IR spectroscopy in the amide I region of the spectra. The spectrum of native GFP is shown as a control.

Endogenous transition metals can bind amyloid peptides, like Aβ42, promoting their aggregation and the formation of amyloid fibers [29]. We analyzed if this pro-aggregating effect can be monitored using the above-described approach. Purified and Gu·HCl denatured Aβ42*wt*-GFP IBs were allowed to refold in PBS in the absence and in the presence of Ca^2+^, Cu^2+^, Fe^3+^, Mg^2+^, Na^+^, Ni^2+^ and Zn^2+^. A highly significant decrease of GFP activity was observed in the presence of the divalent cations Cu^2+^, Ni^2+^ and Zn^2+^ (Figure 2A). This result validates the method since there are strong evidences that zinc and copper enhance amyloid aggregation of Aβ42 and are a component of the senile plaques of Alzheimer's disease patients [30]. In the case of nickel, despite being a metal that lacks physiological relevance, it has also been described to bind Aβ42 and enhance the peptide cytotoxicity, via nanoscale oligomer formation, with the same potency than Cu^+2^[29]. Neither Zn^+2^ nor Cu^+2^ quenched the fluorescence of native untagged GFP (Figure 2B). Moreover, although the presence of Zn^+2^ and Cu^+2^ reduced untagged GFP fluorescence recovery, its effect was clearly lower than the one exerted on the refolding of Aβ42*wt*-GFP IBs (Figure 2B), indicating that in both cases the Aβ42 peptide is a main player in the observed metal promoted aggregation. We analyzed the presence and morphology of aggregates in refolding solutions in the presence and absence of Zn^+2^ and Cu^+2^ by Transmission Electron Microscopy (Figure 3A). In contrast to intact IBs, which appear as electrodense spherical individual entities, all the aggregates in refolding solutions had an amorphous morphology. Nevertheless, Fourier Transformed Infrared Spectroscopy (FT-IR) analysis of the secondary structural features of the aggregated material shows that, in all the cases, the spectra in the amide I region is dominated by a band at 1620–1625 cm-1, typically attributed to the presence of intermolecular β-sheet, which is accompanied by a minor band at 1690 cm-1 corresponding to the splitting of the main β-sheet signal (Figure 3B). These two bands are considered a hallmark of the presence of amyloid-like contacts. The spectra of these aggregates are significantly different from that of native GFP, in which these signatures are absent (Figure 3B).

**Figure 4:**
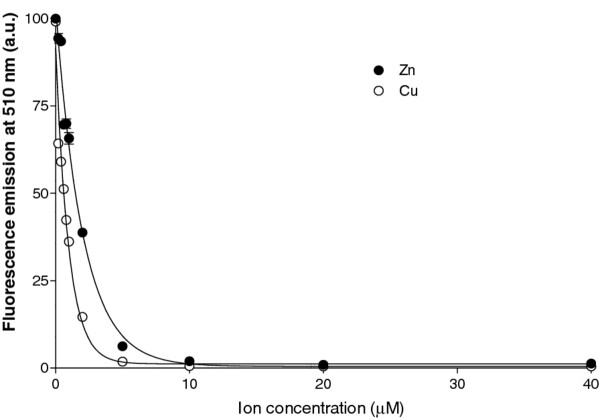
**Effect of metallic ion concentrations on fluorescence recovery.** Aβ42*wt*-GFP IBs were denatured in 8 M Gu·HCl for 4 h and the relative GFP fluorescence recovery upon refolding in the presence of increasing concentrations of Cu^+2^ and Zn^+2^ was monitored at 510 nm.

We explored if the approach allows visualizing a concentration dependent effect of Zn^+2^ and Cu^+2^ on the aggregation of the target at cation concentrations in the range of the physiological levels in human brain [31]. As shown in Figure 4, the approach is highly sensitive to metal concentrations. The titration curves indicate that the impact of Cu^+2^ on aggregation is somehow higher than that of Zn^+2^. Curve fitting to one site binding equation renders apparent dissociation constants of 0.6 and 1.9 μM for copper and zinc, respectively. These data are in good agreement with early reports stating that, despite the two cations bind to equivalent sites in the Aβ peptide, the dissociation constant for copper (0.4 μM) is lower than that of zinc (1.2 μM), as measured by fluorescence and H-NMR at pH 7.2 [32]. Interestingly, despite our assay is not intended for calculating dissociation constants, the ratio between the copper and zinc binding values is also ~ 3. Overall, the approach provides a fast qualitative assessment of metals effect on protein aggregation.

### Identification of inhibitors of metal-triggered Aβ42 aggregation

**Figure 5:**
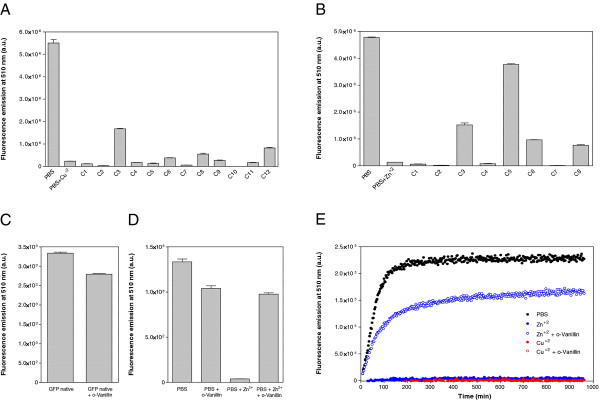
**Fluorescence recovery in the presence of small chemical compounds.** Aβ42*wt*-GFP IBs were denatured in 8 M Gu·HCl and diluted 100-fold in PBS in the absence or presence of 25 μM Cu^+2 ^**(A)** and Zn^+2 ^**(B)** and in the presence of the following small chemical compounds at 25 μM final concentration: azure C (C1), basic blue 41 (C2), meclocycline sulfosalicylate (C3), hemin chloride (C4), *o*-Vanillin (C5), quercetin (C6), congo red (C7), thioflavin –T (C8), apigenin (C9), nordihydroguaiaretic acid (C10), myricetin (C11) and curcumin (C12). **(C)** Quenching of native untagged GFP fluorescence by 25 μM *o*-Vanillin. **(D)** Comparative effect of the absence or presence of 25 μM *o*-Vanillin and/or Zn^+2^ on GFP fluorescence recovery upon dilution of denatured Aβ42*wt*-GFP IBs. **(E)** GFP fluorescence recovery kinetics upon dilution of denatured Aβ42*wt*-GFP IBs in PBS (black solid circles) and PBS with 25 μM Cu^+2^ (red) or Zn^+2^ (blue) ions in the absence (solid circles) or presence (empty circles) of 25 μM *o*-Vanillin.

The identification of small molecules able to interfere protein aggregation is one of the approaches towards therapeutic treatment in amyloid disorders [30,31]. In principle, the outlined assay could be used to screen for such compounds. In particular, in the present work we focused in validating the approach for the identification of inhibitors of copper and zinc promoted aggregation. Despite divalent chelating molecules would work *in vitro*, we discarded the study of this type of molecules since *in vivo* they have shown to sequester cofactors that are essential for the cell physiology [32]. Instead, as a test case, the IBs refolding assay was performed in the presence of selected concentrations of a collection of small compounds that have been reported previously to bind synthetic amyloid Aβ peptides or to modulate their aggregation and/or toxicity [33-37] but have never been assayed before in the presence of metals. The chemical formulae of the different compounds are shown in Table 1. Among the twelve tested compounds only meclocycline sulfosalicylate promoted a significant change in the final levels of GFP fluorescence in the presence of cooper (Figure 5A). This compound was also active in the presence of zinc but the strongest effect in the presence of this cation was observed for *o*-Vanillin (2-Hydroxy-3-methoxybenzaldehyde) (Figure 5B). *o*-Vanillin has a cyclic structure that might quench GFP fluorescence. Effectively, the presence of 25 μM concentration of the compound quenched 15 % of the native untagged GFP fluorescence (Figure 5C). We monitored the effect of *o*-Vanillin on the refolding of Aβ42*wt*-GFP IBs in the absence of metals. The compound did not exhibit any positive effect on GFP recovery by itself and again a 17 % decrease in final fluorescence, mostly attributable to quenching, was observed. Overall, these data indicate that the presence of the compound reduces the metal-promoted aggregation effect by more than 15 fold, allowing to recover about 95% of the GFP-fluorescence observed in the absence of zinc and presence of *o*-Vanillin ( 5D). Interestingly, the *o*-Vanillin effect seems to be specific for zinc, with a negligibly effect for copper. This result is in agreement with previous data indicating that zinc and copper Aβ42 induced aggregation pathways differ in the nature of their intermediate species and suggest that the natural product *o*-Vanillin targets specifically zinc promoted misfolding intermediates, which are characterized by a larger exposition of hydrophobic residues relative to those promoted by copper [38].

**Table 1:** Chemical structure of the small chemical compounds used in the present study

	**Compound**	**Formula**
**C1**	Azure C	
**C2**	Basic blue 41	
**C3**	Meclocycline sulfosalicylate	
**C4**	Hemin	
**C5**	*o*-Vanillin	
**C6**	Quercetin	
**C7**	Congo Red	
**C8**	Thioflavin T	
**C9**	Apigenin	
**C10**	Nordihydroguaiaretic acid	
**C11**	Myricetin	

Although, to our knowledge, no *in vivo* effects of *o*-Vanillin on Aβ42 promoted neuronal toxicity have been reported so far (work in progress). A closely related compound differing only in a CH_2_ group, 2-Hydroxy-3-ethoxybenzaldehyde, completely blocked the neurotoxicity of the peptide to rat hippocampal neurons in culture [39], indicating that despite the simplicity of our assay, it may identify physiologically relevant hit compounds.

To obtain further insights on the effects of copper, zinc and *o*-Vanillin on Aβ42 aggregation, we monitored the kinetics of GFP refolding after IBs denaturation in the presence and absence of these molecules by following the changes in fluorescence emission (Figure 5E). In PBS, GFP fluorescence was recovered following a double exponential curve with a rate constant of 0.90 ± 0.02 s^-1^ and a half-life of 46.21 min for the fast reaction phase. The presence of both copper and zinc abrogated completely the fluorescence recovery already at the beginning of the refolding reaction, likely indicating that they promote a very fast aggregation of the fusion protein that totally competes the GFP domain folding reaction. The presence of *o*-Vanillin has a negligible effect on copper containing solutions. In contrast, this molecule allows recovery of 70 % of the fluorescence at the end of the reaction in the presence of zinc. The rate constants and half-life for the fast phase were very close to those exhibited in the absence of metals, with values of 0.87 ± 0.03 s^-1^ and 48.29 min, respectively. This indicates that this compound acts interfering with zinc promoted Aβ42 aggregation without affecting GFP folding. Interestingly, the GFP fluorescence recovery reaction is completed after 3.5 h, being thus a faster assay than those relying on the aggregation of synthetic peptides, which usually require at least overnight incubation [40]. We used the metallochromic Zincon reagent [41] to quantify the free levels of Zn^2+^ and Cu^2+^ in the absence and presence of *o*-Vanillin using spectrophotometry. No differences in free ion metal levels were observed (data not shown) suggesting that the compound does not act as a chelator but rather affects the refolding/aggregation kinetics of misfolded GFP fusions.

## Conclusions

**Figure 6:**
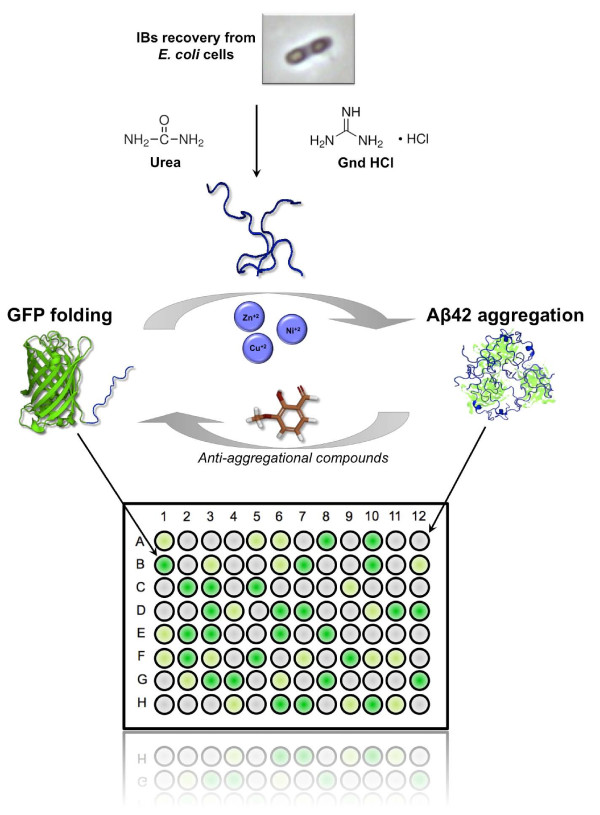
**Outline of the screening method to identify molecules able to modulate amyloid aggregation.** The method is based on the kinetic competition between the aggregation promoted by the Aβ42 moiety and the folding of the GFP domain after denaturation and refolding of Aβ42*wt*-GFP IBs. Molecules that accelerate the aggregation reaction result in low fluorescence recovery and *vice versa.*

Based in our previous knowledge on the amyloid-like nature of the IBs formed by Aβ peptides [16,23] and the *in vivo* correlation between the aggregation rates and the total IBs activity [22,26], we describe here a straightforward approach to identify compounds that modulate Aβ aggregation using bacterial IBs. The method is implemented using 96 well plates and the reaction takes less than four hours, making it suitable for high-throughput screening (Figure 6). Because most amyloid proteins and peptides form IBs when expressed in bacteria [17], the approach may have, in principle, a broad applicability in the search for aggregation modulators in conformational disorders. The assay does not require a detailed understanding of the structure of the aggregating species, and can provide an unbiased method for the discovery of hit compounds. IBs can be produced and purified in large amounts, making the method cost-effective, especially when compared with the use of synthetic peptides. Despite its simplicity, the approach allows to distinguish between aggregation pathways and to identify inhibitors with therapeutic potential.

## Methods

### Production and purification of inclusion bodies

*Escherichia coli* BL21DE3 competent cells were transformed with pET28 vectors (Novagen, Inc., Madison, WI, USA) encoding the sequences for Aβ42*wt*-GFP fusion and the mutant Aβ42F19D-GFP, as previously described [25].

10 mL of bacterial cultures were grown at 37°C and 250 rpm in LB medium containing 50 μg/mL of kanamycin. At an OD_600_ of 0.5, 1 mM of isopropyl-β-D-1-thiogalactopyranoside (IPTG) was added to induce recombinant protein expression.

After 4 hours, cells were harvested by centrifugation and pellets were re-suspended in lysis buffer (100 mM NaCl, 1 mM EDTA and 50 mM Tris pH 8) to purify intracellular inclusion bodies (IBs), as previously described [41]. Briefly, protease inhibitor PMSF and lysozyme were added at the final concentrations of 15 mM and 300 μg/mL, respectively. After incubating at 37°C for 30 min, detergent NP-40 was added at 1 % and cells were incubated at 4°C for 50 min under mild agitation. To remove nucleic acids, cells were treated with DNase and RNase at 15 μg/mL at 37°C for 30 min. IBs were collected by centrifugation at 12,000x*g* for 10 min and washed with lysis buffer containing 0.5 % Triton X-100. Finally, they were washed three times with PBS to remove remaining detergent.

### *In vitro* refolding assay

15 μL of purified IBs at OD_360_ = 10 were centrifuged for 10 min at 12000x*g*. To denature the aggregates, the pellets were re-suspended in 10 μL of 8 M Gu·HCl or 10 M urea and incubated at room temperature for 4 h. For the refolding process, denatured aggregates were dissolved in 990 μL of refolding buffer. These buffers were based on PBS, previously treated with Chelex 100 chelating resin from Sigma-Aldrich (St. Louis, MO, USA), and the following salts and compounds according to the different refolding assays: CaCl_2_, FeCl_3_, MgCl_2_, NaCl, NiCl_2_, ZnCl_2_, CuCl_2_, apigenin, azure C, basic blue 41, congo red, curcumin, hemin chloride, meclocycline sulfosalicylate, myricetin, nordihydroguaiaretic acid, *o*-Vanillin (2-hydroxy-3-methoxybenzaldehyde), thioflavin -T and quercetin, all obtained from Sigma-Aldrich (St. Louis, MO, USA). Equimolar concentrations of purified untagged GFP were used in control experiments. GFP fluorescence of the solutions containing refolded IBs or untagged GFP were measured in a 96 well plate in a Victor 3 Plate Reader (Perkin-Elmer, Inc., Waltham, MA, USA) using excitation and emission wavelength filters of 405 nm and 510 nm, respectively or in a Jasco FP-8200 spectrofluorometer using excitation and emission wavelengths of 480 nm and 510 nm, respectively. Measurements were performed in triplicate. For kinetic experiments, the refolding step was followed using the same parameters and reading the fluorescence emission every 2 min for 16 h. In order to homogenize the samples, these were briefly shacked (for 5 s) before each determination.

### Transmission electronic microscopy

IBs or aggregates containing solutions were placed on carbon-coated copper grids, and left to stand for five minutes. The grids were washed with distilled water and stained with 2 % (w/v) uranyl acetate for another two minutes before analysis using a HitachiH-7000 transmission electron microscope (Hitachi, Tokyo, Japan) operating at accelerating voltage of 75 kV.

### Secondary structure determination

Aggregates present in refolding solutions were precipitated by centrifugation at 12.000 xg (g en cursiva i sense espais) for 30 min, resuspended in Milli-Q water and analyzed, together with purified untagged GFP, by FT-IR spectroscopy using a Bruker Tensor 27 FT-IR Spectrometer (Bruker Optics Inc) with a Golden Gate MKII ATR accessory. Each spectrum consists of 16 independent scans, measured at a spectral resolution of 2 cm^-1^ within the 1700–1500 cm^-1^ range. All spectral data were acquired and normalized using the OPUS MIR Tensor 27 software.

## Competing interests

The authors declare that they have no competing interests.

## Authors’ contributions

SV supervised the project, designed the study and drafted the manuscript. AVP and AE carried out all experiments and drafted the manuscript. RS and NSG critically revised and corrected the manuscript. All authors read and approved the final manuscript.
